# Challenges facing primary health care: a perspective from Colombia’s Caribbean region

**DOI:** 10.3389/fpubh.2025.1681840

**Published:** 2025-10-09

**Authors:** Ana L. Ríos García, María Y. Expósito Concepción, Rafael Tuesca-Molina, Diana C. Consuegra Cabally, Tania Acosta, Allison E. Cano Barrios

**Affiliations:** Department of Public Health, Universidad del Norte, Colombia, Barranquilla, Colombia

**Keywords:** primary health care, family health, mental health, vulnerable populations, healthpolicy

## Abstract

**Introduction:**

Primary Health Care (PHC) has been a central focus in health policy debates, nationally and internationally. In this context, efforts aim to achieve an effective integration between PHC and the broader health system to ensure equitable, accessible, and needs-based care aligned with the real needs of the population.

**Objective:**

To describe the characteristics of the families participating in the study and to conduct a theoretical-reflective analysis of the challenges faced by PHC, as well as the responses required from service providers and local health authorities. The approach emphasizes family and community health to better meet the needs of vulnerable families in the Colombian context.

**Methodology:**

A cross-sectional descriptive study was conducted. The study included 417 families, totaling 1,923 individuals. Instruments used included the Family Health Diagnosis form and the Family APGAR. A descriptive analysis of frequency and percentage was performed for qualitative variables. Bivariate analysis was used to explore associations between family functionality (functional vs. dysfunctional) and a range of sociodemographic, economic, and life-event variables. Prevalence ratios (PR) with 95% confidence intervals (CI) were calculated, and chi-square tests (χ^2^) were applied to determine statistical significance (*p* < 0.05).

**Results:**

Adults accounted for one-third of the population, followed by youth (15%) and children (16%), together comprising 67%. Smaller proportions were observed in early childhood and older adults. Most individuals (72%) are covered under the subsidized health regime, in keeping with the study’s focus on vulnerable populations. According to the Family APGAR, 57% of families exhibit some degree of dysfunction. Only one-third of individuals participate in health promotion and maintenance programs appropriate to their life stage. Less than 1% use mental health services (psychology or psychiatry), and 27% are unaware of or do not attend any such programs.

**Conclusion:**

The findings underscore the urgent need to strengthen PHC as the coordinating axis of family care, moving beyond a model centered exclusively on clinical and individual attention. A comprehensive perspective that addresses social and emotional determinants of health is essential to respond effectively to the needs of vulnerable families.

Primary health care has become a key strategy for addressing health issues in highly vulnerable contexts. In Colombia, Law 1,438 of 2011 positioned it as the top priority and a highly cost-effective approach to improving the population’s health conditions (1).

It is important to highlight that primary care goes beyond basic services, as it promotes intersectoral collaboration to address the social determinants of health, fosters community empowerment as a fundamental factor to ensure the sustainability and cultural appropriateness of programs, and prioritizes strategies for health promotion and maintenance over those focused solely on curative approaches (1, 2).

Given this reality, the Colombian health system is currently undergoing a reform process aimed at addressing long-standing challenges with limited progress, such as universal access to health services, efficiency, reducing care fragmentation, and implementing an effective policy for the training and development of human resources in health (3).

In Colombia, health promotion and maintenance programs are regulated by Resolution 3,280 of 2018 (4), which emphasizes the importance of implementing interventions based on the life-course approach and adapted to the specific characteristics of families. Within this framework, diagnosis is prioritized through family health assessment tools, which are key instruments for comprehensive health risk management (5, 6).

In the family health approach, the family is considered a fundamental structure for the comprehensive development of the human being. This approach values aspects such as the integration of services, intersectoral actions, and especially the pillar of caregiving, with an emphasis on the emotional dimension. The family represents the first point of human contact, made up of its members as well as the environment that surrounds them (7). During the COVID-19 pandemic, people were exposed to high levels of fear and anxiety, resulting from both the disease and family losses. These emotions can be overwhelming and lead to intense reactions in both adults and children (8, 9).

In this context, a community intervention is being implemented in the Colombian Caribbean Region to address the post-pandemic effects of COVID-19 on mental health. It targets vulnerable families, specifically in Barranquilla, Soledad, and Cartagena (10). The purpose of this article is to present, based on the characterization of the target family groups, a theoretical and reflective analysis on the challenges of primary health care and the responses that both service providers and local health authorities must offer, within the framework of a family and community health approach to address the needs of vulnerable families in the Colombian context.

## 2 Methods

This study is part of the project approved under Call No. 918–2022, “Strengthening regional capacities for public health research,” issued by Minciencias through contract No. 587–2022. It corresponds to a mixed-methods study (qualitative-quantitative) conducted in two phases. The first phase focused on exploration and diagnosis, and the second on the design, implementation, and evaluation of an intervention program between 2022 and 2025. This article presents the results of the first phase, which corresponds to a descriptive cross-sectional study. Based on the findings, the authors conduct a theoretical and reflective analysis of the challenges involved in addressing the needs of vulnerable families in the Colombian context.

### 2.1 Study population

The study was conducted in the Caribbean region of Colombia, specifically in the departments of Atlántico (Barranquilla District and the municipality of Soledad) and Bolívar (Cartagena District), in low socioeconomic status neighborhoods. A convenience and snowball sampling strategy was used. Three clusters were selected one in each district/municipality and previously trained personnel applied data collection instruments in the selected areas.

Families residing in the selected communities, with at least one member over 18 years of age, who voluntarily agreed to participate through informed consent and provided complete information for analysis, were included. Recruitment was carried out through schools and community foundations with an established presence in the study areas. This strategy is essential in community-based research, as it facilitates access and promotes the active participation of families (11, 12). A total of 417 families participated in the study, comprising 1,923 individuals.

The study was conducted in communities where randomization is either impossible or impractical due to limited accessibility to participants, as well as conditions of vulnerability and security. Although this design does not allow for the generalization of findings, the sampling strategy used provides significant value by enabling the selection of cases (individuals, families, contexts, and situations) of particular interest. This enriches both data collection and analysis, and turns the results into a solid foundation for subsequent phases or future studies.

To minimize selection bias, participant recruitment sites were diversified across the three municipalities to enhance coverage. The sample composition was continuously monitored in real time against known distributions (local censuses, SISBEN, etc.). Furthermore, awareness-raising strategies were implemented prior to and during fieldwork to foster trust, comprehension, and a favorable disposition among participants. Safeguards were also adopted to ensure the protection of participants’ rights, dignity, and well-being, particularly considering the involvement of vulnerable populations.

### 2.2 Instruments

The instruments were administered at times agreed upon with participants to avoid fatigue effects and reduce the likelihood of biased responses. Data collection was conducted in two separate sessions, with a minimum interval of 2 days, after obtaining informed consent.

Family APGAR: This instrument was developed by Smilkstein in 1978 to assess family functionality and cohesion (13). It evaluates the perception of family members regarding the overall functioning of their family unit. It consists of five items covering key aspects: Adaptation, Partnership, Growth, Affection, and Resolve. Each item is rated using a five-point Likert scale: never (0), almost never (1), sometimes (2), almost always (3), and always (4), yielding a total score ranging from 0 to 20. For this study, the version adapted for Colombia by Arias et al. was used (14). The interpretation of the results is classified into four levels: Good family functionality (17–20 points), Mild dysfunction (13–16 points), Moderate dysfunction (10–12 points), and Severe dysfunction (9 points or less) (15–17). These studies in Colombia have reported the average internal consistency of the APGAR, with Cronbach’s alpha of 0.90 (16) and 0.793 (15).

This instrument is not limited to measuring an isolated aspect; rather, it assesses key dimensions of family dynamics: adaptation, partnership, growth, affection, and resolution. This approach allows the family to be understood as a social system rather than merely the sum of individuals. It is a brief tool, easy to administer and understand, which facilitates its use in communities with low educational levels or in contexts where time and resources are limited. It enables the early detection of situations of stress, conflict, or lack of social support factors closely associated with physical, mental, and social health problems. Due to its simplicity and validity, it can be applied in descriptive studies, community surveys, or public health interventions, making it a versatile instrument that can be compared across different social and cultural groups. By analyzing family functioning, it is possible to establish associations with social phenomena such as poverty, migration, violence, substance use, or unequal access to services, thereby providing evidence to inform policies and programs. The APGAR has been widely validated in Latin America and in different cultural contexts, which facilitates the comparison of results and strengthens the robustness of findings in social research (14–16). Moreover, according to regulations of the Colombian Ministry of Health, it is the recommended instrument for assessing family functioning in the Colombian context (18).

Family Health Diagnostic Form: This instrument allowed for the socio-family characterization. It was designed based on the characterization form recommended by the Ministry of Health (18) and the version proposed by Ríos et al. (19) in a study conducted in Colombian ethnic communities. In the context of this article, several variables of interest are presented: individual life cycle, family life cycle, family structure, and family crises. Family crises were categorized into two main groups: normative (or developmental) and non-normative (or non-developmental). Normative crises refer to expected and predictable events in the family life cycle, such as marriage or the birth of a child. In contrast, non-normative crises are unexpected and difficult-to-predict events that often cause significant stress, such as job loss or the death of a loved one. The questionnaire inquired about events occurring within the past 6 months, distinguishing between expected (normative) and unexpected (non-normative) crises. It also explored whether these crises were ongoing at the time of the evaluation (20). Additionally, the form collected information regarding the families’ perception of their current situation and access to health programs.

A database was created using Microsoft Excel, where the extracted data were coded and an initial filtering was performed. In IBM SPSS v.29, the variables were analyzed using absolute and relative frequencies in percentages. A bivariate analysis was conducted to identify associations between family functionality (classified as functional or dysfunctional) and various independent variables of sociodemographic, economic, and life event types.

For this analysis, the prevalence ratio (PR) was used as the measure of association, appropriate for cross-sectional studies with frequent outcomes, and 95% confidence intervals (95% CI) were calculated for each estimate. The statistical significance of the associations was assessed using the chi-square test (χ^2^), with a *p*-value < 0.05 considered statistically significant. A reference category was established for each independent variable, from which the PRs were interpreted, allowing the identification of factors potentially associated with a higher or lower prevalence of family dysfunction.

## 3 Results

Table 1 shows that approximately half of the families selected belong to the Cartagena District. The sex ratio was 1.04. One-third of the population falls within the adulthood stage, followed by youth (15%) and childhood (16%), which together represent 67% of the total population; the lowest proportions correspond to old age and early childhood.

**Table 1:** Social and family characterization of participating families and individuals.

Characteristic	Distribution	Frequency and percentage
Families by Municipality	Barranquilla	142 (34.05%)
	Soledad	45 (10.79%)
	Cartagena	230 (55.16%)
Total individuals by sex	Men	941 (48.93%)
	Women	982 (51.06%)
Family Typology	Nuclear	139 (33.33%)
	Extended	150 (35.97%)
	Expanded	18 (4.32%)
	Single-parent	39 (9.35%)
	Reconstituted	53 (12.71%)
	Conjugal dyad	16 (3.83%)
	Single-person	2 (0.48%)
Life Course	Early childhood (0–5 years)	193 (10.03%)
	Childhood (6–11 years)	314 (16.33%)
	Adolescence (12–17 years)	252 (13.10%)
	Youth (18–28 years)	294 (15.29%)
	Adulthood (29–59 years)	691 (35.93%)
	Old age (60 years and over)	161 (8.37%)
	Not reported	18 (0.94%)
Family Life Cycle	Couple formation	6 (1.44%)
	Family expansion - pregnancy / initial childrearing (newborns, infants)	30 (7.19%)
	Family expansion with preschool children	20 (4.80%)
	Consolidation – family with school-age children	81 (19.42%)
	Consolidation – family with adolescent children	106 (25.42%)
	Launching stage (“launching platform”)	152 (36.45%)
	Post-parental family – middle-aged	11 (2.64%)
	Post-parental family – older adults	11 (2.64%)
Health Insurance Scheme	Contributory	465 (24.18%)
	Subsidized	1,386 (72.07%)
	Special	13 (0.68%)
	Uninsured	45 (2.34%)
	No response (Not reported)	14 (0.73%)
Migrant Status	No	1771 (92.10%)
	Yes	152 (7.90%)
Family APGAR (Functionality)	Good function	258 (43%)
	Mild dysfunction	138 (23%)
	Moderate dysfunction	69 (11.6%)
	Severe dysfunction	134 (22.4%)

Colombian Caribbean Region (Departments of Atlántico and Bolívar). 2023–2025. Source: Data collected by the research team.

A total of 72% reported being affiliated with the subsidized health insurance regime, which aligns with the study’s focus on vulnerable populations. Approximately 2 to 3% reported no affiliation or did not respond; this may be related to the 8% identified as having a migrant condition or status.

Regarding the family life cycle, approximately 60% were in the consolidation stage, with school-age children or adolescents, and a smaller proportion in the couple formation stage. In terms of family typology, one-third were classified as extended families, followed by nuclear families (33.33%). Lower proportions were observed in conjugal dyads (3.8%) and single-person households (0.48%; Table 1).

Regarding the application of the Family APGAR, 57% of families showed some degree of dysfunction. These findings reflect a significant proportion of families experiencing difficulties in their internal dynamics, which may be related to the presence of both normative and non-normative crises. Additionally, family type influences available resources, dynamics, and potential challenges.

Table 2 presents information on health status and access to programs and/or procedures in accordance with Resolution 3,280 of 2018 in Colombia, which promotes health promotion and maintenance actions throughout the life course. In this regard, one-third of the population reported attending such programs.

**Table 2:** Perceptions, conditions, and access to health programs among the families in the study.

Characteristic	Distribution	Frequency and percentage
Self-reported Disability	None (No report)	1713 (89.08%)
	Visual	71 (3.69%)
	Mental	45 (2.34%)
	Motor	18 (0.93%)
	Multiple	20 (1.04%)
	Speech	12 (0.62%)
	Others	30 (1.56%)
	No response	4 (0.20%)
Self-reported symptoms of depression and anxiety	None (No report)	1809 (94.07%)
	Depression	62 (3.22%)
	Anxiety	33 (1.71%)
	Both	15 (0.78%)
	No response	4 (0.21%)
Access to health maintenance, promotion, and care programs	Life course promotion and maintenance programs (Child growth and development, adolescent, youth, adult, older adults)	657 (34.17%)
	General Medicine	406 (21.11%)
	Two or more programs/services	296 (15.39%)
	Specialty care (Pediatrics, Internal Medicine, Dermatology)	29 (1.50%)
	Mental Health (Psychology or Psychiatry)	13 (0.67%)
	Not applicable (Does not know)	520 (27.04%)
	No response	2 (0.10%)

Colombian Caribbean Region (Departments of Atlántico and Bolívar). 2023–2025. Source: Data collected by the research team.

Less than 1% reported accessing mental health programs (psychology or psychiatry services), and 27% either do not attend or are unaware of any services aimed at health promotion and maintenance.

A total of 89% of participants reported not having a disability. Among reported disabilities, visual impairments accounted for approximately 4%, followed by mental disabilities at 2.3%. These figures are consistent with self-reported symptoms related to depression and anxiety, which were close to 5%.

In Table 3, a low incidence of crises such as retirement and pregnancy is observed as concurrent factors, with occurrence percentages below 5% in the past 6 months. In contrast, illness appears with a notable frequency (14%) and significant persistence (20%), which could indicate a prolonged impact on the families studied. Natural death and the arrival of a new family member in the last 6 months show intermediate frequencies (11 and 8%, respectively), with persistence ranging between 4 and 6%, suggesting that their impact was more prolonged.

**Table 3:** Normative crises reported by participants’ families in the past 6 months.

Normative crisis	Response	Frequency	Percentage (%)
Natural death	No	355	85.0
	Yes	46	11.0
	Ongoing	16	4.0
Illness	No	276	66.0
	Yes	58	14.0
	Ongoing	83	20.0
Arrival of a new family member	No	359	86.0
	Yes	33	8.0
	Ongoing	25	6.0
Starting school	No	371	89.0
	Yes	27	6.0
	Ongoing	19	5.0
Retirement	No	411	98.5
	Yes	1	0.3
	Ongoing	5	1.2
Pregnancy	No	393	94.0
	Yes	16	4.0
	Ongoing	8	2.0

Colombian Caribbean Region (Departments of Atlántico and Bolívar). 2023–2025. Source: Data collected by the research team.

These findings suggest considerable variability in the relevance of different normative crises. The high persistence of illness highlights its potential to generate prolonged imbalances within families. Information on the persistence of each event provides a crucial temporal dimension for understanding the dynamics and impact of these normative crises in the analyzed context.

Table 4 shows that, in the past 6 months, participating families reported different types of non-normative crises. Violent death was infrequent, with 6% of families having experienced it and only 1% reporting that the situation persisted. Regarding couple separation, 6% reported having gone through it recently, and 3% indicated it was still ongoing. Leaving the home (referring to unplanned departures due to situations such as abuse, unresolved conflicts, etc.) was reported by 5.1%, while 4.8% indicated the situation remains active.

**Table 4:** Non-normative crises in the past six months among participating families.

Non-normative crises	Has presented the mentioned crisis	Frequency	Percentage
Violent death	No	389	93%
	Yes	26	6%
	Ongoing	5	1%
Separation	No	378	91%
	Yes	25	6%
	Ongoing	14	3%
Leaving home	No	376	90.1%
	Yes	21	5.1%
	Ongoing	20	4.8%
School expulsion	No	408	97.8%
	Yes	5	1.2%
	Ongoing	4	1%
Unemployment	No	221	53%
	Yes	67	16%
	Ongoing	129	31%
Couple problems	No	331	79%
	Yes	39	9%
	Ongoing	47	11%
Economic changes	No	210	50%
	Yes	98	24%
	Ongoing	109	26%
Adoption	No	413	99%
	Yes	3	0.7%
	Ongoing	1	0.3%
Infidelity	No	400	95.9%
	Yes	8	1.9%
	Ongoing	9	2.2%
Addiction in any family member	No	338	81%
	Yes	32	8%
	Ongoing	47	11%
COVID-19 in the nearby community	No	352	84%
	Yes	62	15%
	Ongoing	3	1%
COVID-19 in any family member	No	353	85%
	Yes	63	15%
	Ongoing	1	—
Death of a family member due to COVID-19	No	336	80.5%
	Yes	31	7.5%
	Ongoing	1	0.3%
	No answer	49	11.7%

Caribbean Region of Colombia (Departments of Atlántico and Bolívar). 2023–2025. Source: Data collected by the research team.

School expulsion was a rarely reported event, affecting only 1.2% of families, with 1% where this crisis persists. In contrast, unemployment showed a higher incidence: 16% had experienced it recently, and 31% stated that the issue is still ongoing, highlighting it as one of the most critical situations.

Relationship problems were present in 9% of families, with 11% reporting they are still ongoing. Economic changes were also significant, with 24% having experienced them and 26% where they persist, indicating a prolonged impact on family stability. Other events such as adoption (0.7%) and infidelity (1.9% recent incidence and 2.2% persistence) were less common. However, addiction in a family member was reported by 8%, and in 11% the problem is still ongoing.

Regarding the pandemic, 15% reported having had COVID-19 cases in the nearby community, with 1% where the impact remains; similar figures were recorded for cases within the family (15%), although persistence was minimal. Death from COVID-19 affected 7.5%, and a small percentage (0.3%) reported that the situation continues to have consequences.

In the last variable (death of a family member due to COVID-19), it is noteworthy that 49 families did not answer the question, and that several of the non-normative crises experienced by families still persist meaning people perceive them as ongoing. The coexistence of multiple crises, especially when they persist over time, represents a risk factor for family balance and the well-being of its members.

Statistically significant associations were identified between family dysfunction and various sociodemographic variables and life events (Supplementary Figure S1).

Regarding educational level, individuals with secondary education showed a lower probability of family dysfunction compared to those with no formal education (PR = 0.777; 95% CI: 0.621–0.973; *p* = 0.028). Although not all educational levels showed significant differences, a general trend was observed in which higher levels of education were associated with lower prevalence of family dysfunction. It is not possible to categorically state that all higher levels of education exert the same protective effect. Contextual factors such as the quality of education received, socioeconomic inequities, or the influence of other social determinants are likely to modulate this relationship and help explain the lack of significance at certain educational levels.

Marital status showed a significant association with family functionality. Single individuals were at a considerably higher risk of family dysfunction compared to those who were married (PR = 1.786; 95% CI: 1.351–2.360; *p* = 0.000), suggesting that the absence of a stable marital relationship may limit the emotional and structural resources that promote family cohesion. Similarly, unemployment was significantly associated with higher levels of dysfunction (PR = 1.186; 95% CI: 1.027–1.370; *p* = 0.020), reinforcing the role of economic factors as key determinants of family well-being. Couple relationship problems also showed a strong association with family dysfunction (PR = 1.406; 95% CI: 1.230–1.608; *p* = 0.000), highlighting the impact of affective relationship quality on family system stability. Taken together, these findings emphasize that both emotional instability and adverse economic conditions can compromise family functioning, weakening bonds and reducing the perceived emotional support among its members.

Recent economic changes were significantly associated with a higher prevalence of family dysfunction (PR = 1.154; 95% CI: 1.004–1.327; *p* = 0.044), suggesting that financial instability may generate stressors that disrupt family dynamics and reduce adaptive capacity. In a similar vein, pregnancy was also linked to increased levels of family dysfunction (PR = 1.285; 95% CI: 1.041–1.585; *p* = 0.019), possibly due to the emotional, economic, and relational adjustments required during this life stage, particularly in contexts marked by vulnerability or limited support networks.

Regarding health conditions, a strong and significant association was identified between visual disability and family dysfunction (PR = 0.458; 95% CI: 0.297–0.708; *p* = 0.000). Families in which a member had a visual impairment reported lower levels of functionality, which may reflect the additional care demands, emotional burden, and systemic challenges that affect cohesion and mutual support within the household. These findings highlight how both situational stressors (such as economic fluctuations or pregnancy) and chronic conditions (like disability) can undermine family functioning by imposing sustained or acute pressures on family roles, resources, and relationships.

## 4 Discussion

The characterization conducted with a family health approach highlights the needs of individuals and families in maintaining their health. When examining how these needs are addressed through national health promotion and maintenance programs, it becomes evident that some remain unmet. However, if the Colombian health system adopted a Primary Health Care (PHC) approach, one that integrates intersectoral collaboration, emphasizes health promotion and disease prevention programs, and fosters community empowerment, and then all identified needs could be adequately addressed.

The majority of the families participating in the study are in the launching and consolidation phases with adolescent children. This finding is relevant, as these stages of the family life cycle are typically characterized by greater adaptive demands, stemming both from the changes inherent to adolescence and from the preparation of children for independent living. In these phases, families face challenges related to the progressive autonomy of adolescents, the reconfiguration of intrafamily relationships, and the need to balance support with the promotion of independence.

Therefore, identifying a high proportion of families in these phases implies recognizing a scenario that requires interventions aimed at strengthening parenting skills, promoting healthy communication dynamics, and providing institutional support to facilitate the transition to later stages of the life cycle with a lower risk of deterioration in family functionality (4, 21).

The health insurance scheme shows that the population is predominantly enrolled in the subsidized system. This finding reflects the socioeconomic vulnerability of the participating families, as this scheme is intended for individuals with lower income and limited payment capacity. From a public health perspective, this pattern of insurance may have implications for the timeliness, quality, and continuity of access to services, particularly in areas such as mental health and family care, where gaps in coverage and resource availability persist (4, 5, 8).

The results also highlight the complexity of current family dynamics and the importance of addressing them with a comprehensive PHC approach. Both normative and non-normative crises, whether newly occurring or ongoing, directly affect the emotional, social, and economic stability of households and, therefore, their functionality (21, 22).

Among the results, the low level of family access to mental health services stands out; it is not only statistically significant but also deeply concerning in social and human rights terms. It falls below the national average, where only about 1.56% have access and more than 60% of those in need do not receive care. This reflects sharp regional inequities, due to the urban concentration of services and the structural marginalization of certain populations and regions of the country. The situation is even more serious than in many developing countries, where access is also limited but rarely falls below 10% (21, 23). This finding underscores the urgent need to prioritize public policies, infrastructure, workforce training, and the removal of barriers (geographic, cultural, economic, and stigma-related) to mental health care in vulnerable populations.

Previous studies have documented that enrollment in the subsidized health insurance scheme is associated with greater geographical, administrative, and cultural barriers to effective access to, which could negatively affect families ‘ability to cope with crises or to maintain an adequate level of functionality (24, 25).

One of the most relevant findings is the high frequency of family dysfunction, which may be related to structural factors such as unemployment, economic changes, and relationship problems, all non-normative crises with a considerable emotional burden. Many of these situations are ongoing and represent a challenge for health services, as they cannot be resolved solely through clinical care but require a multidisciplinary, continuous, and family-centered approach (22, 26).

In the analysis of family functioning with social determinants, reverse causality cannot be ruled out; that is, pre-existing family dysfunction may hinder the resolution of crises, leading to their persistence within the family unit. Future research, preferably with a longitudinal design, is needed to clarify the directionality of this relationship.

Moreover, the COVID-19 pandemic exposed and worsened many of these crises, causing losses, infections, and unresolved grief in a significant proportion of families. These events, though specific to a recent context, reveal the vulnerability of support networks and the need to strengthen emotional and community components of healthcare (21, 26, 27).

The results of the family and social characterization of the study population reveal a diverse composition in terms of family types, life cycles, and life courses. This diversity presents multiple challenges for the healthcare system, particularly within the framework of Primary Health Care (PHC) in Colombia. Such diversity demands responses that are adapted and sensitive to the social, cultural, and territorial contexts in which families live.

Various studies have shown that health systems based on the principles of primary care, conceived as continuous, comprehensive, integrated, and coordinated care, achieve better outcomes and greater health equity compared to systems centered on the traditional biomedical model (28, 29). Strengthening Integrated Health Service Networks (RISS) is equally important. This includes ensuring care for a defined population, the need for healthcare institutions with different levels of care and complexity, and the geographic scope in which they would be implemented (30).

This characterization highlights that PHC, as a key strategy within the health system, must move toward more inclusive and preventive models that prioritize family mental health promotion, early crisis intervention, and education for family life. To achieve this, it is essential to have teams trained in the family approach, sufficient time for community work, and tools that not only allow for diagnosis but also support family processes effectively (31–33).

## 5 Challenges

The data obtained reveal not only the segmentation of care but also the limited response capacity of the system. This underscores the importance of addressing the issue from a comprehensive perspective. In this regard, the challenges outlined are not presented as definitive conclusions of the study, but rather as constructive contributions that may serve as a framework for action to help address the identified weaknesses and strengthen the delivery of health services.

In summary, family dysfunction is associated with emotional, social, and economic factors. Higher educational attainment serves as a protective factor, while unemployment, being single, couple conflicts, pregnancy, economic changes, and visual disability increase the risk of dysfunction. These findings highlight the need for comprehensive interventions that strengthen family resilience and address both structural and relational vulnerabilities.

In this context, Primary Health Care (PHC), in its role as the first level of contact with the population, faces the challenge of expanding its scope beyond the individual to effectively incorporate the family-centered approach. This implies not only identifying dysfunctions but also intervening in a timely and contextualized manner, considering the crises families are experiencing and their cumulative impact. The persistence of events such as illness, unemployment, or addictions, for example, requires sustained interventions, coordination with other sectors, and close support, often limited by the fragmentation of services and high care demand (34). This presents a significant challenge for the new health reform proposed in the Colombian context.

In this sense, the health system becomes one of the biopsychosocial determinants of health, since PHC promotes equal access to services, care proportional to needs, and joint work with other sectors to address the root causes of health problems across different age groups. Therefore, a key factor in this context is for intersectoral actions to be assumed by local governments, allowing for more effective and contextually appropriate responses in each community (24, 35–37).

Additionally, the health system must strengthen primary care with psychosocial teams, train health professionals in community mental health, and integrate brief interventions into first-level services (38). The National Mental Health Policy (2024–2030) offers a favorable framework, but its effective implementation still faces significant logistical and budgetary challenges (39). In this regard, aspects related to the timeliness and accessibility of health services require macro and meso level management policies (from the ministry and local health departments) and programs and activities to be developed at the micro level (in local health services) (40).

In addition, the presence of migrant and displaced populations in these vulnerable communities highlights the need to adapt health services to attend to mobile populations, many of whom lack documentation or have irregular status (41). Policies for migrants in the country must continue to be strengthened to ensure a culturally competent PHC approach.

Likewise, it is essential to design and implement differentiated family care strategies that consider the high prevalence of non-traditional family structures. These strategies should include specific interventions in caregiving dynamics, emotional support, and health-related decision-making (42–48).

As a limitation of the study, the descriptive nature of the results should be noted, and it is acknowledged that the use of non-probabilistic sampling in a cross-sectional study with families in conditions of vulnerability restricts statistical representativeness and, consequently, the generalization of the findings. Likewise, the presence of social desirability bias cannot be ruled out, since some responses may have been influenced by the intention to present a more favorable situation than the actual one. Nevertheless, the study retains its value for describing local realities, making inequities visible, and generating hypotheses for future studies with more robust probabilistic designs.

Therefore, given the method employed, the aim is not to establish or favor causal inferences; rather, the potential findings should be further investigated in studies with greater methodological rigor, broader representativeness, and larger sample sizes.

To mitigate biases in the interpretation of the results, various methodological strategies were implemented. First, clear inclusion and exclusion criteria were defined, ensuring coherence in the selection of the study population. Second, data collection was carried out using standardized instruments administered by previously trained researchers, which reduced variability in survey administration and interviewer influence. In addition, stratified analyses were conducted by sociodemographic variables and municipality, allowing for the identification of differential patterns and preventing homogeneous conclusions that could obscure inequities. Triangulation with secondary sources strengthened the consistency of the findings, while transparent reporting of limitations contributed to a critical and contextualized interpretation. Finally, collective and multidisciplinary review of the results reduced the risk of biased interpretations, fostering a more integral and balanced approach.

Overall, the proposed approach promotes a holistic and interdisciplinary perspective, which is key to strengthening processes of care, support, and empowerment for families and communities in conditions of vulnerability.

## 6 Conclusion

Family dysfunction in this sample from the Colombian Caribbean was associated with socioeconomic factors (unemployment, relationship problems, economic changes, and educational level), marital status (particularly singlehood), and certain personal factors such as pregnancy and visual impairment. Although these associations suggest that structural and relational conditions may influence family functioning, it is important to note that they do not necessarily imply causality. These 368 findings highlight the importance of designing comprehensive and multisectoral interventions that address both the emotional and socioeconomic dimensions of family life, with the aim of fostering resilience and strengthening family systems.

The observed health situation reveals limitations in access to and utilization of the health promotion and maintenance programs established under Resolution 3,280 of 2018. Significant gaps were identified in the use of these services, particularly in relation to mental health, reflecting the persistence of barriers to comprehensive care throughout the life course. Likewise, the presence of disability conditions and symptoms associated with emotional problems was noted; although reported in a smaller proportion, these issues demand priority attention from a preventive perspective. These findings underscore the need to strengthen Primary Health Care through comprehensive, intersectoral, and sustained strategies that promote early detection, health education, and equitable access to services, with the aim of fostering individual, family, and community well-being.

In the studied sample, it was observed that certain family crises, such as retirement and pregnancy, presented a low recent incidence, whereas events such as illness had a more frequent and prolonged impact on family dynamics. Other events, such as natural death or the arrival of a new family member, showed an intermediate incidence but with effects that extended over time, suggesting that their influence on family functioning may be more sustained. These findings reflect the variability in the ways different life events affect families and highlight the importance of adopting support strategies that take into account both the frequency and the duration of their impacts on family life.

Despite the multiple challenges faced by Primary Health Care in Colombia, significant progress and strengths in its implementation can be recognized. The country continues to uphold the right to health and universal access as inalienable principles. In this context, it is essential to resume and strengthen social, economic, and environmental policies, as well as to rebuild scenarios that support a fairer, more equitable, and people-centered health model.

## Data Availability

The original contributions presented in the study are included in the article/Supplementary material, further inquiries can be directed to the corresponding author.
