# Supplementary material for: Challenges facing primary health care: a perspective from Colombia’s Caribbean region
Source: Front Public Health. 2025 Oct 9;13:1681840. doi: 10.3389/fpubh.2025.1681840 (PMC12546244; doi:10.3389/fpubh.2025.1681840)
Supplement: Supplementary file 1 [file Table_1.DOCX]

Supplementary Material

# Supplementary Figures and Tables

**Supplementary Table S1.** Family functioning and socio-family characteristics. Caribbean Region of Colombia (Departments of Atlántico and Bolívar). 2023–2025

| Variable | Categories | Functional | | Dysfunctional | | Total | Sig | RP* | IC 95% | | SIG** |
| --- | --- | --- | --- | --- | --- | --- | --- | --- | --- | --- | --- |
|  |  | **n** | **%** | **n** | **%** |  |  |  | **Inf** | **Sup** |  |
| City | Barranquilla y área met. | 96 | 37% | 113 | 33% | 209 | 0,301 | 1 |  |  |  |
|  | Cartagena | 162 | 63% | 228 | 67% | 390 |  | 1,081 | 0,930 | 1,257 | 0,308 |
| Gender | Male | 61 | 24% | 97 | 28% | 158 | 0,187 | 1 |  |  |  |
|  | Female | 197 | 76% | 244 | 72% | 441 |  | 0,901 | 0,776 | 1,046 | 0,173 |
| Education level | None | 16 | 6% | 33 | 10% | 49 | 0,009 | 1 |  |  |  |
|  | Primary | 56 | 22% | 110 | 32% | 166 |  | 0,984 | 0,787 | 1,230 | 0,887 |
|  | Secondary | 132 | 51% | 145 | 43% | 277 |  | 0,777 | 0,621 | 0,973 | 0,028 |
|  | Technical | 40 | 16% | 43 | 13% | 83 |  | 0,769 | 0,579 | 1,023 | 0,071 |
|  | University | 14 | 5% | 10 | 3% | 24 |  | 0,619 | 0,371 | 1,032 | 0,066 |
| Marital status | Married | 48 | 19% | 32 | 9% | 80 | <,001 | 1 |  |  |  |
|  | Separated | 15 | 6% | 14 | 4% | 29 |  | 1,207 | 0,760 | 1,917 | 0,426 |
|  | Cohabiting | 112 | 43% | 104 | 30% | 216 |  | 1,204 | 0,890 | 1,628 | 0,229 |
|  | Single | 76 | 29% | 190 | 56% | 266 |  | 1,786 | 1,351 | 2,360 | 0,000 |
|  | Widowed | 7 | 3% | 1 | 0% | 8 |  | 0,313 | 0,049 | 1,993 | 0,219 |
| Migrant population | No | 239 | 93% | 305 | 89% | 544 | 0,180 | 1 |  |  |  |
|  | Yes | 19 | 7% | 36 | 11% | 55 |  | 1,167 | 0,950 | 1,434 | 0,141 |
| Health system affiliation (SGSSS) | Contributory | 62 | 24% | 75 | 22% | 137 | 0,812 | 1 |  |  |  |
|  | Subsidized | 186 | 72% | 253 | 74% | 439 |  | 1,053 | 0,886 | 1,250 | 0,558 |
|  | Special | 1 | 0% | 3 | 1% | 4 |  | 1,370 | 0,763 | 2,461 | 0,292 |
|  | Unaffiliated | 9 | 3% | 10 | 3% | 19 |  | 0,961 | 0,611 | 1,512 | 0,865 |
| Family life cycle | Couple formation | 4 | 2% | 2 | 1% | 6 | 0,019 | 1 |  |  |  |
|  | Expansion | 23 | 9% | 37 | 11% | 60 |  | 1,850 | 0,586 | 5,837 | 0,294 |
|  | Consolidation | 106 | 41% | 175 | 51% | 281 |  | 1,868 | 0,600 | 5,814 | 0,281 |
|  | Launching | 115 | 45% | 110 | 32% | 225 |  | 1,467 | 0,469 | 4,583 | 0,510 |
|  | Post-parenting | 10 | 4% | 17 | 5% | 27 |  | 1,889 | 0,587 | 6,074 | 0,286 |
| Family structure | Nuclear | 88 | 34% | 108 | 32% | 196 | 0,87 | 1,000 |  |  |  |
|  | Extended | 100 | 39% | 132 | 39% | 232 |  | 1,033 | 0,872 | 1,223 | 0,710 |
|  | Augmented | 8 | 3% | 15 | 4% | 23 |  | 1,184 | 0,856 | 1,637 | 0,308 |
|  | Single-parent | 18 | 7% | 21 | 6% | 39 |  | 0,977 | 0,712 | 1,342 | 0,887 |
|  | Blended | 33 | 13% | 54 | 16% | 87 |  | 1,126 | 0,916 | 1,386 | 0,260 |
|  | Couple dyad | 10 | 4% | 10 | 3% | 20 |  | 0,907 | 0,575 | 1,432 | 0,676 |
|  | Single-person | 1 | 0% | 1 | 0% | 2 |  | 0,907 | 0,226 | 3,649 | 0,891 |
| Disability | None | 236 | 91% | 315 | 92% | 551 | 0,517 | 0,572 | 0,532 | 0,615 | 0,000 |
|  | Motor | 3 | 1% | 1 | 0% | 4 |  | 0,250 | 0,046 | 1,365 | 0,109 |
|  | Auditory | 1 | 0% | 4 | 1% | 5 |  | 0,800 | 0,516 | 1,240 | 0,318 |
|  | Visual | 13 | 5% | 11 | 3% | 24 |  | 0,458 | 0,297 | 0,708 | 0,000 |
|  | Mental | 3 | 1% | 5 | 1% | 8 |  | 0,625 | 0,365 | 1,069 | 0,086 |
|  | Other | 1 | 0% | 2 | 1% | 3 |  | 1,000 | 1,000 | 1,000 | 1,000 |
|  | Multiple | 1 | 0% | 2 | 1% | 3 |  | 0,667 | 0,300 | 1,484 | 0,321 |
|  |  | 0 | 0% | 1 | 0% | 1 |  | 1 |  |  |  |
| Alcohol use | No | 208 | 81% | 282 | 83% | 490 | 0,447 | 1 |  |  |  |
|  | Yes | 50 | 19% | 57 | 17% | 107 |  | 0,926 | 0,763 | 1,123 | 0,433 |
|  |  | 0 | 0% | 2 | 1% | 2 |  | 1,738 | 1,610 | 1,875 | 0,000 |
| Violent death | No | 236 | 91% | 319 | 94% | 555 | 0,335 | 1 |  |  |  |
|  | Si | 22 | 9% | 22 | 6% | 44 |  | 0,870 | 0,642 | 1,179 | 0,369 |
| Natural death | No | 231 | 90% | 291 | 85% | 522 | 0,129 | 1 |  |  |  |
|  | Yes | 27 | 10% | 50 | 15% | 77 |  | 1,165 | 0,972 | 1,396 | 0,099 |
| Illness | No | 188 | 73% | 230 | 67% | 418 | 0,153 | 1 |  |  |  |
|  | Yes | 70 | 27% | 111 | 33% | 181 |  | 1,115 | 0,965 | 1,288 | 0,141 |
| Separation | No | 240 | 93% | 305 | 89% | 545 | 0,130 | 1 |  |  |  |
|  | Yes | 18 | 7% | 36 | 11% | 54 |  | 1,191 | 0,973 | 1,459 | 0,091 |
| Leaving home | No | 240 | 93% | 308 | 90% | 548 | 0,241 | 1 |  |  |  |
|  | Yes | 18 | 7% | 33 | 10% | 51 |  | 1,151 | 0,928 | 1,428 | 0,201 |
| New family member arrival | No | 226 | 88% | 295 | 87% | 521 | 0,696 | 1 |  |  |  |
|  | Yes | 32 | 12% | 46 | 13% | 78 |  | 1,042 | 0,853 | 1,272 | 0,690 |
| Starting school | No | 221 | 86% | 299 | 88% | 520 | 0,468 | 1 |  |  |  |
|  | Yes | 37 | 14% | 42 | 12% | 79 |  | 0,925 | 0,742 | 1,152 | 0,484 |
| School expulsion | No | 254 | 98% | 331 | 97% | 585 | 0,268 | 1 |  |  |  |
|  | Yes | 4 | 2% | 10 | 3% | 14 |  | 1,262 | 0,900 | 1,772 | 0,178 |
| Unemployment | No | 131 | 51% | 140 | 41% | 271 | ,018^*^ | 1 |  |  |  |
|  | Yes | 127 | 49% | 201 | 59% | 328 |  | 1,186 | 1,027 | 1,370 | 0,020 |
| Relationship problems | No | 222 | 86% | 242 | 71% | 464 | <,001^*^ | 1 |  |  |  |
|  | Yes | 36 | 14% | 99 | 29% | 135 |  | 1,406 | 1,230 | 1,608 | 0,000 |
| Retirement | No | 253 | 98% | 338 | 99% | 591 | 0,3 | 1 |  |  |  |
|  | Yes | 5 | 2% | 3 | 1% | 8 |  | 0,656 | 0,267 | 1,608 | 0,357 |
| Economic changes | No | 144 | 56% | 162 | 48% | 306 | ,044^*^ | 1 |  |  |  |
|  | Yes | 114 | 44% | 179 | 52% | 293 |  | 1,154 | 1,004 | 1,327 | 0,044 |
| Pregnancy | No | 247 | 96% | 313 | 92% | 560 | 0,052 | 1 |  |  |  |
|  | Yes | 11 | 4% | 28 | 8% | 39 |  | 1,285 | 1,041 | 1,585 | 0,019 |
| Adoption | No | 255 | 99% | 336 | 99% | 591 | 0,749 | 1 |  |  |  |
|  | Yes | 3 | 1% | 5 | 1% | 8 |  | 1,099 | 0,640 | 1,889 | 0,732 |
| Infidelity | No | 247 | 96% | 325 | 95% | 572 | 0,802 | 1 |  |  |  |
|  | Yes | 11 | 4% | 16 | 5% | 27 |  | 1,043 | 0,757 | 1,437 | 0,797 |
| Family member with addictions | No | 205 | 79% | 250 | 73% | 455 | 0,081 | 1 |  |  |  |
|  | Yes | 53 | 21% | 91 | 27% | 144 |  | 1,150 | 0,990 | 1,336 | 0,067 |
| COVID-19 in the community | No | 209 | 81% | 293 | 86% | 502 | 0,106 |  |  |  |  |
|  | Yes | 49 | 19% | 48 | 14% | 97 |  | 0,848 | 0,684 | 1,050 | 0,131 |

*PR Prevalence ratio, ** Chi Square
